# Polypharmacy in older adults: a narrative review of definitions, epidemiology and consequences

**DOI:** 10.1007/s41999-021-00479-3

**Published:** 2021-03-10

**Authors:** Farhad Pazan, Martin Wehling

**Affiliations:** Clinical Pharmacology Mannheim, Medical Faculty Mannheim, Ruprecht-Karls-Heidelberg University, Theodor-Kutzer-Ufer 1-3, 68167 Mannheim, Germany

**Keywords:** Polypharmacy, Multimorbidity, Older adults, Health outcomes, Inappropriate prescribing

## Abstract

**Background:**

The number of older adults has been constantly growing around the globe. Consequently, multimorbidity and related polypharmacy have become an increasing problem. In the absence of an accepted agreement on the definition of polypharmacy, data on its prevalence in various studies are not easily comparable. Besides, the evidence on the potential adverse clinical outcomes related to polypharmacy is limited though polypharmacy has been linked to numerous adverse clinical outcomes. This narrative review aims to find and summarize recent publications on definitions, epidemiology and clinical consequences of polypharmacy.

**Methods:**

The MEDLINE database was used to identify recent publications on the definition, prevalence and clinical consequences of polypharmacy using their respective common terms and their variations. Systematic reviews and original studies published between 2015 and 2020 were included.

**Results:**

One hundred and forty-three definitions of polypharmacy and associated terms were found. Most of them are numerical definitions. Its prevalence ranges from 4% among community-dwelling older people to over 96.5% in hospitalized patients. In addition, numerous adverse clinical outcomes were associated with polypharmacy.

**Conclusion:**

The term polypharmacy is imprecise, and its definition is yet subject to an ongoing debate. The clinically oriented definitions of polypharmacy found in this review such as appropriate or necessary polypharmacy are more useful and relevant. Regardless of the definition, polypharmacy is highly prevalent in older adults, particularly in nursing home residents and hospitalized patients. Approaches to increase the appropriateness of polypharmacy can improve clinical outcomes in older adults.

## Key summary points

**Aim:**

This narrative review aims to find and summarize recent publications on definitions, epidemiology and clinical consequences of polypharmacy.

**Findings:**

Numerous definitions of polypharmacy and associated terms were found. The prevalence of polypharmacy greatly varies, and numerous adverse clinical outcomes were associated with polypharmacy.

**Message:**

The clinically oriented definitions of polypharmacy are more useful and relevant. Regardless of the definition, polypharmacy is highly prevalent in older adults. Approaches to increase the appropriateness of polypharmacy can improve clinical outcomes in older adults.

## Introduction

Multimorbidity is highly prevalent in our aging societies and it often leads to the use of multiple medications in older patients [1–5]. There are also a number of other factors which are associated with an increased use of drugs in older people such as the wider and growing availability of effective medications in developed countries, and the numerous guidelines that encourage the use of multiple drugs for the management of common diseases [4, 6, 7]. While there is still no consensus on what should be regarded as polypharmacy, there are ongoing efforts to improve its definition and shift the focus from the mere number of drugs to their appropriateness, effects and, ultimately, to related clinical outcomes in older patients [6, 8]. These efforts of course need to be backed by evidence.

In this context, it is important to understand that the process of biological aging is often accompanied by changes in pharmacokinetics and pharmacodynamic in older people [9]. Consequently, there should have been more efforts towards clinical testing of medications specifically in older adults. Regrettably, an exclusion of older adults from clinical approval trials is often observed that has led to a lack of evidence regarding the safety and efficacy of many medications in this population [10, 11]. Thus, the appropriateness of many drugs in an increasing number of older multimorbid patients remains still undetermined. Inevitably, this lack of evidence has often led to inappropriate drug treatment and, consequently, to various adverse clinical outcomes. This large-scale issue was also recognized by the World Health Organization and led to the initiation of the ongoing patient safety campaign called “The third Global Patient Safety Challenge: Medication without harm” with “the goal of reducing severe, avoidable drug-related harm worldwide by 50%” until 2022 [12].

Therefore, to differentiate between appropriate and inappropriate drug treatment numerous listing tools/approaches (prescribing appropriateness assessment tools and criteria) have been proposed [4].

In this work, we searched for the existing definitions of polypharmacy, its prevalence among older adults in various clinical settings and evidence for its clinical consequences in geriatric patients.

## Search strategy

This work represents a narrative review that provides an overview of the recent publications on polypharmacy. It is focused on definition, epidemiology, and outcomes of polypharmacy in older adults. For this purpose, we used MEDLINE to search for related publications. The search terms were the following: (polypharmacy OR multiple medication* OR multiple medicine* OR multiple drug* OR Polypharmacy [Mesh] OR many medication* OR definition of polypharmacy OR prevalence of polypharmacy or epidemiology of polypharmacy OR consequences of polypharmacy OR outcomes of polypharmacy). The following filters were used to narrow our search: free full text, full text, review, systematic review, publication date: from November 2015 to November 2020, Humans, English, Aged: 65+ years, 80 and over: 80+ years. In addition, the period for our search was limited to the past 5 years because we aimed to find the most recent literature which would add to the results from the existing reviews also included in this narrative review. All papers found in MEDLINE were assessed for eligibility by title and abstract screening. In addition to the database search, we performed manual searches from the reference lists of some selected articles.

### Definition of polypharmacy

We found that there is no generally accepted definition for polypharmacy. This fact has also been acknowledged in a recent report from the World Health Organization (WHO) which stated that:

“Polypharmacy is the concurrent use of multiple medications. Although there is no standard definition, polypharmacy is often defined as the routine use of five or more medications. This includes over-the-counter, prescription and/or traditional and complementary medicines used by a patient” [12].

Certainly, the concurrent intake of five or more drugs is the most common definition of polypharmacy in the literature [13]. Nevertheless, about 143 definitions of polypharmacy and associated terms exist according to a systematic review of definitions of polypharmacy [14] and other more recent publications [7, 8, 11, 15, 16]. The vast majority of those (*n* = 112) are mere numerical definitions, meaning that only the number of drugs is used to check for the presence of polypharmacy in an individual [14]. These numerical definitions are very heterogenous and partly include associated terms such as minor [17–19], mild [20], moderate [21], major [19, 22–25] and excessive polypharmacy [24, 26–29] to characterize the severity of polypharmacy [14]. Besides, the threshold for these numerical definitions of polypharmacy ranges from 2 or more to 11 or more drugs [14] and the cut-off/range for the associated terms partly varies as well. For instance, moderate polypharmacy is defined as the daily intake of 4–5 medication in one source [14] and as the intake of 5–9 drugs in another [21].

In addition, there are 11 numerical definitions of polypharmacy which also classify the duration of drug treatment, about 4 numerical definitions which also include the health care setting and 16 descriptive definitions of polypharmacy [8, 14–16, 30]. However, the few numerical definitions including the health care setting can be regarded as only numerical definitions which were used in a specific clinical setting. Categories [14] and examples for various definitions of polypharmacy and associated terms are depicted in Table 1. Examples for the more recent definitions of polypharmacy have been underlined in this table. Other new definitions of polypharmacy included unnecessary polypharmacy, and polypharmacy of unclear benefit [8].

**Table 1:** Categories and examples for the existing definitions of polypharmacy and associated terms (examples of the more recent definitions of polypharmacy have been underlined)

Numerical only definitions	Numerical definitions including a duration of therapy	Numerical definitions including a health care setting	Descriptive definitions
Five or more medications [27]	Two or more medications for over 240 days [23]	Five or more medications at hospital discharge [31]	Necessary polypharmacy: “Necessary polypharmacy regimens should be considered additional medications that can optimize functional status and prevent disability in older adults. For older patients receiving NP regimens, the benefits outweigh the risks” [8]
Six or more medications [32]	Over five medications for 90 or more days [33]	Five to nine medications during hospital stay [34]	Qualitative polypharmacy: “prescription of five or more medications including at least one drug considered potentially inappropriate for older adults” [15]
Ten or more medications [14]	Five to nine medications for 90 or more days [35]	Ten or more medications during hospital stay (also called excessive polypharmacy) [34, 36]	Psychotropic polypharmacy: “the concurrent use of two or more psychotropic agents in one individual” [16]
Seven or more medications [37]	Five or more medications in the same quarter of a year [38]	–	Appropriate polypharmacy: “optimization of medications for patients with complex and/or multiple conditions where medicine usage agrees with best evidence” [14, 39]
Five to nine medications [40]	Five or more medications in the same month [41]	–	“Use of medications which are not clinically indicated” [14, 42]

### Epidemiology

The prevalence of polypharmacy found in the literature greatly varies. It ranges from around 4% to about 96.5% depending on the age group, definition, healthcare setting and region [7, 11].

A cross-sectional analysis [43] from “wave 6” of the Survey of Health, Ageing, and Retirement in Europe (SHARE) database including data from 34,232 participants (mean age: 75.1 ± 7.2 years) revealed that the prevalence of polypharmacy, defined as the concurrent use of five or more medications, in older community-dwelling older adults (aged 65 years or more) ranges from 26.3 to 39.9% across 17 European countries plus Israel [43]. It is noteworthy that the medication use in this study was self-reported and a high number of older adults with comorbidities might have been excluded [43].

The overall prevalence of polypharmacy across all countries was 32.1% (95% CI 31.5–32.7). Besides, the lowest prevalence of polypharmacy was found in Switzerland (26.3%; 95% CI 25.8–26.8), Croatia (27.3%; 95% CI 26.8–27.9) and Slovenia (28.1%; 95% CI 27.6–28.6), while Portugal (36.9%; 95% CI 36.3–37.5), Israel (37.5%; 95% CI 36.9–38.2) and the Czech Republic (39.9%; 95% CI 39.3–40.5) had the highest prevalence of polypharmacy [43].

**Fig. 1:**
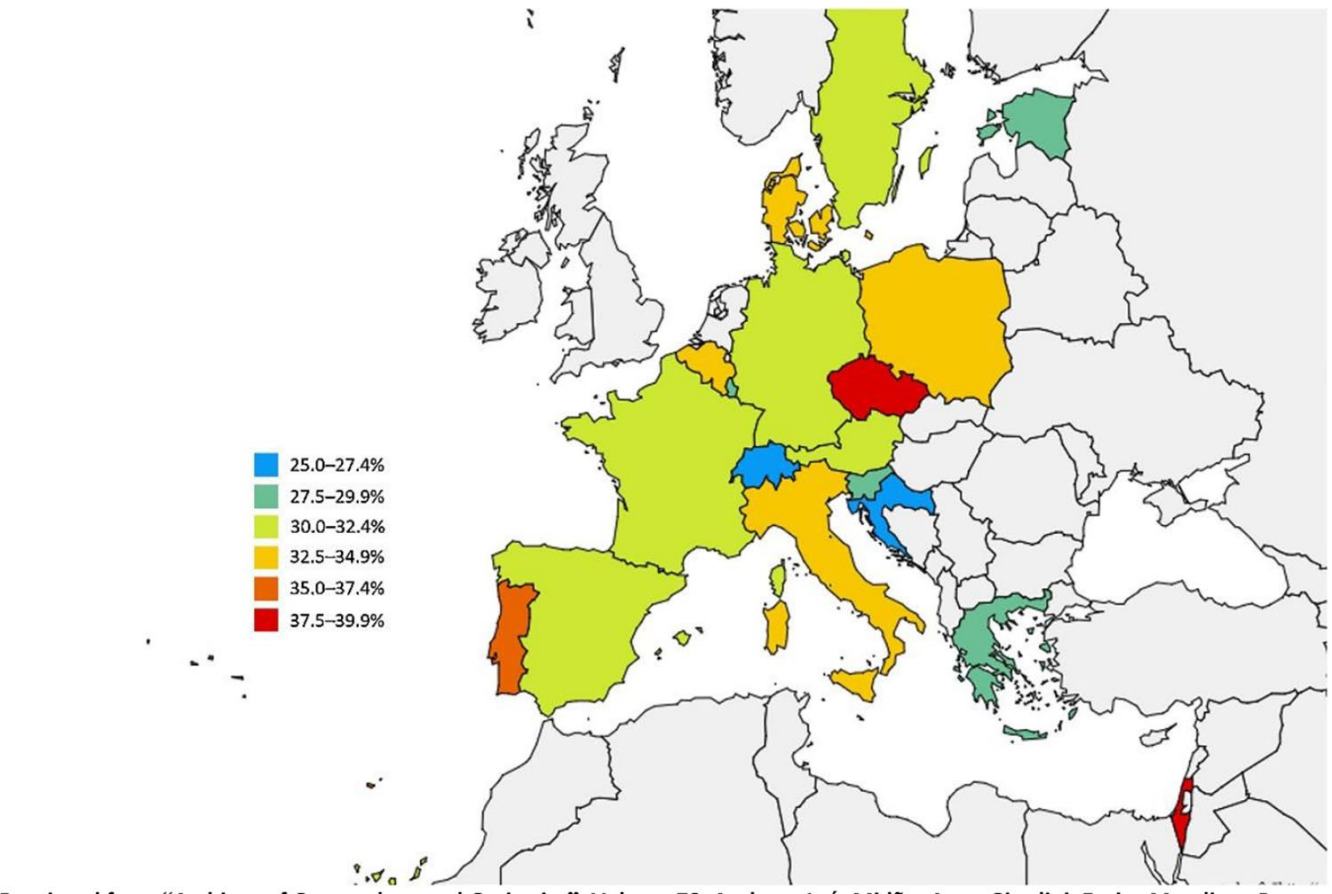
Prevalence of polypharmacy (defined as the concurrent use of five or more medications per day) in older adults across 17 European countries and Israel according to “wave 6” of the Survey of Health, Ageing, and Retirement in Europe (SHARE) [43] (Reprinted from Midão et al. [43], Copyright (2018), with permission from Elsevier. https://doi.org/10.1016/j.archger.2018.06.018)

The prevalence rates were also standardized by three age groups: 65–74, 75–84 and 85 years or older. As anticipated, in higher age groups the overall prevalence of polypharmacy was also greater. The overall prevalence for each age group was as follows: 25.3% (95% CI 24.6–26.0) for participants aged 65–74, 36.4% (95% CI 35.4–37.5) for participants aged 75–44 and 46.5% (95% CI 44.6–48.4) for participants aged 85 years or older [43]. The overall prevalence of polypharmacy was almost identical for women (32.1%; 95% CI 31.3–32.9) and men (32.2%; 95% CI 31.4–33.0) [43]. The prevalence of polypharmacy across 17 European countries and Israel according to Midão et al. [43] is depicted in Fig. 1.

Based on the publications from the European Union funded “Stimulating Innovation in the Management of Polypharmacy and Adherence in the Elderly (SIMPATHY) project” [44, 45] the prevalence of polypharmacy (defined as taking ten or more medications) using population-level data from “The Information Services Division (ISD), a division of National Services Scotland” [46], ranged from approximately 6–36% in older adults. According to this data, deprivation had a profound impact on the prevalence of polypharmacy in all age groups. For instance, in people aged 65–69 the prevalence of polypharmacy was about 24% in the most deprived vs. around 7% in the least deprived [45]. In people aged 85 or older, the prevalence of polypharmacy for the two most deprived group of participants (around 33% in the most deprived and about 30% in the second most deprived) was unexpectedly lower as compared to the younger counterparts aged 80–84 (around 36% in the most deprived and about 31% in the second most deprived) [44, 45].

The analysis of the population-level data from a prospective survey investigating the biological and genetic determinants of cardiovascular disease in the population of Lausanne (CoLaus study, Switzerland) revealed that 25.5% of people aged from 65 to 81 years regularly used five or more medications [47]. Another study based on claims data from the largest health insurance in Switzerland revealed that 41.2% of older adults concurrently received 5 or more medications [48].

In a repeated cross-sectional large-scale study (between 338,025 and 539,752 older individuals) from Ireland using electronic data from pharmacy claims over 15 years (1997–2012), the prevalence of polypharmacy (five or more regular prescription medications) increased from 17.8% in 1997 to 60.4% in 2012. In addition, excessive polypharmacy (ten or more regular prescription medications) showed a similar trend. It increased from 1.5 to 21.9% in people aged 65 years or older [49].

In a cross-sectional analysis of adult electronic primary healthcare records from Scotland, the prevalence of polypharmacy (the use of 4–9 medications) was 28.6% in adults aged 60–69 years and 51.8% in those aged 80+ years [50]. In this study, the term polypharmacy was not clearly stated. Instead, the “higher” consumption of drugs was categorized into either using 4–9 medications or ten or more. In addition, the prevalence of patients taking ten or more medications was 7.4% in people aged 60–69 years and 18.6% in those aged 80+ years [50].

Another repeated cross-sectional analysis of community-dispensed prescribing data in the Tayside region of Scotland showed that the use of ten or more drugs in older adults more than tripled from 4.9% in 1995 to 17.2% in 2010 [51]. In the same time period, the use of five or more medications was also increased by twofold from around 20% to around 40% among older adults aged 65–69 years old [51].

In the U.S., population-level data from the National Health and Nutrition Examination Survey (NHANES) revealed a significant increase in the prevalence of polypharmacy (five or more prescription medications) from 24% (95% CI 21–26) to 39% (95% CI 35–44) among older adults between 1999/2000 and 2011/2012 [52].

In a large observational cohort study including 2057 older emergency department patients from Ancona in Italy [53]. The prevalence of polypharmacy (the concomitant use of 6–9 drugs in the last 3 months) was 30.3% and excessive polypharmacy (the concomitant ten or more drugs in the last 3 months) was observed in 17.8% of the patients [53].

A large (*N* = 1,742,336) prospective, longitudinal, register-based cohort study specifically analyzed the epidemiology of polypharmacy in older adults between 2010 and 2013 in Sweden [54]. This study showed that the prevalence of polypharmacy (5+ drugs) was 44% and 11.7% of the individuals included had excessive polypharmacy (used ten or more drugs) [54]. The incidence rate of newly developed polypharmacy was 19.9 per 100 person-years, and the incidence rate of excessive polypharmacy was 8.0 per 100 person-years [54].

A longitudinal study from The Netherlands including data from elderly patients visiting general practices in Groningen between 1994 and 1997 showed a prevalence of “major polypharmacy” (long-term use of six or more drugs; long-term was defined as “for over 240 days in a year” [23]) in only 4% of cases at the end of 1997. Besides, at the end of the study 9% of the participants were prescribed 4–5 drugs (long-term) which was considered as moderate polypharmacy and 28% of the patients 2–3 drugs (long-term) defined as minor polypharmacy in this study [23]. Over the course of four years, about 20% of older people developed polypharmacy in this population [23].

According to another study based on the Korea Health Insurance Review and Assessment Service—National Patient Sample (HIRA-NPS) data from 2010 and 2011, polypharmacy (six or more medications) was found in 86.4% of older people in Korea [24]. Major polypharmacy (11 medications or more) and excessive polypharmacy (21 medications or more) was observed in 44.9% and 3% of cases each [24].

A longitudinal observational study from Taiwan, which included frail Taiwanese older adults with long-term care needs also revealed a high prevalence of polypharmacy (prescribed with five or more drugs) in about 84% of cases [24]. However, persistent polypharmacy (polypharmacy for 181 days or more) was observed in only 31% of cases [55]. Another study from Taiwan, which used Taiwan’s Longitudinal Health Insurance Database to assess drug use for older adults showed that polypharmacy (use of five or more drugs) was present in 28.2% of cases [56].

In the GLISTEN (Gruppo di Lavoro Italiano Sarcopenia—Trattamento e Nutrizione; Italian working group on sarcopenia—nutrition and treatment) study, the prevalence of polypharmacy (using five or more medications daily) in patients admitted to geriatric and internal medicine acute care ward of 12 Italian hospitals was 70.2%. In the same cohort, the prevalence of hyper-polypharmacy (using ten or more medications daily) was 13% [57]. Another study in older hospitalized patients (aged 60 or over) from India showed that 45% of the patients presented with polypharmacy (used 5–9 medications) and 45.5% with high-level polypharmacy (used ten or more drugs) [58]. According to a cross-sectional study in older (aged 80 years or older) hospitalized patients from China, the prevalence of “hyper-polypharmacy” (11 or more drugs) was 96.5% [59].

In a cross-sectional analysis on nursing home residents participating in the “Services and Health for Elderly in Long TERm care” (SHELTER) project the prevalence of polypharmacy (5–9 drugs) and excessive polypharmacy (ten or more drugs) were 49.7% and 24.3% each [60]. Other studies assessing medication use in nursing home residents reported a prevalence of polypharmacy (five or more drugs) between 38.1 and 91.2% [61]. In addition, the prevalence of polypharmacy defined as the use of ten or more drugs ranged between 10.6 and 65% [61].

### Clinical consequences of polypharmacy

Polypharmacy has been linked to numerous negative clinical outcomes such as frailty, hospitalization and even mortality [62, 63]. Here, we report the results from previous reviews and recent studies on the association between polypharmacy and major clinical outcomes in older adults.

#### Polypharmacy and frailty

While polypharmacy and frailty are highly prevalent in older adults the causal relationship between them is still unclear [11]. According to a recent systematic review and meta-analysis [64] which investigated the cross-sectional association between polypharmacy/hyper-polypharmacy and the presence of prefrailty/frailty as well as the risk of incident prefrailty/frailty in adults with polypharmacy, a strong and bidirectional association between both polypharmacy and hyper-polypharmacy and frailty was found [64]. This study indicated that 75% of adults with polypharmacy are pre-frail/frail. Besides, only a few longitudinal studies on the risk of incident prefrailty/frailty in adults with baseline polypharmacy were found [64]. In a meta-analysis of three of those studies, a significantly higher odd of developing prefrailty in robust persons was found in the presence of polypharmacy [64]. In a longitudinal study [65], taking seven or more medications was associated with a 2.5 increased risk of developing frailty over 8 years [64]. This systematic review concluded that more research is needed to assess the causal relationship between polypharmacy and frailty [64]. Another systematic review indicated that polypharmacy could play a major role in the development of frailty and stated that the causal relationship is uncertain and appears to be bidirectional [66]. Besides, a cohort study of 772 Spanish older adults showed that polypharmacy is associated with death, incident disability, hospitalization, and emergency department visits in frail and prefrail older adults, but not in robust older people [67].

#### Polypharmacy and death

A systematic review and meta-analysis by Leelakanok et al. revealed a significant association between mortality and polypharmacy [68]. Besides, when polypharmacy was defined categorically, a dose–response relationship was seen across escalating thresholds (namely 1–4 medications, five medications, 6–9 medications and ten or more medications) [68]. It is important to mention that in patients with polypharmacy the risk of mortality may also be increased by the presence of more chronic conditions. Hence, confounding by indication has to be addressed when assessing the relationship between polypharmacy and mortality [62]. For instance, in a study by Schöttker et al. addressing this issue, polypharmacy was not independently associated with non-cancer mortality after adjustment for chronic conditions and by using propensity score matching [62, 69, 70]. Conversely, in a recent nationwide longitudinal cohort study from Korea, polypharmacy was associated with a higher risk of all-cause death, even after adjustment for comorbidities and propensity-score matching [71]. In addition, a danish nationwide population-based cohort study showed an association between increasing number of medications and mortality. In brief, for each extra medication the mortality increased by over 3% in the fully adjusted model which included diseases and Barthel Index [72].

#### Polypharmacy and hospitalization

Several studies in community-dwelling older adults [73] and nursing home residents [74] have shown an association between polypharmacy and hospitalization [11, 62]. The association has been shown for any hospitalization, unplanned hospitalization, and re-hospitalization in hospital-based samples [62]. For instance, a longitudinal health insurance database study from Taiwan investigating the association between polypharmacy and all-cause and fracture-specific admission to hospital [56], showed an independent association between polypharmacy and all-cause and fracture-specific hospitalization [56]. In another study including nursing home residents from Australia, polypharmacy (defined as nine or more regular medications) was associated with time to the first hospitalization, number of hospitalizations and hospital days per person-year [75]. Furthermore, in a large observational cohort study in a geriatric hospital, polypharmacy (having 6–9 drug prescriptions in the last 3 months) and excessive polypharmacy (ten or more drug prescriptions in the last 3 months) were both associated with emergency department revisit and hospital admission [53]. In the aforementioned nationwide longitudinal cohort study from Korea polypharmacy was associated with a higher risk of hospitalization as well [71].

#### Polypharmacy and falls

Numerous studies have shown an association between polypharmacy and falls [62, 76]. For example, a register-based study from Sweden confirmed the association between polypharmacy and risk of falling [77]. However, an adjustment for fall-risk-inducing drugs (FRIDs) weakened this association [77]. In a longitudinal study from England, the rate of falls was 21% higher in older adults with polypharmacy (five or more drugs) compared with people without polypharmacy [78]. Using a lower threshold of four or more drugs for polypharmacy, the rate of falls was 18% higher in people with polypharmacy compared with people without [78]. Hyper-polypharmacy (ten or more drugs) was associated with a 50% higher rate of falls [78]. In another study, the number of fall-risk medications was associated with fall-related hospital admissions though polypharmacy could not be identified as an independent risk factor [79]. Recently, a prospective cohort study also revealed that polypharmacy is associated with an increased falls risk in UK care home residents [80].

#### Polypharmacy and cognitive impairment

Polypharmacy and specially psychotropics and anticholinergic drugs have been associated with cognitive impairment in various studies [62, 81].

For example, in a 12-year longitudinal register-based study from South Korea, polypharmacy was associated with the development of dementia [82]. Another longitudinal study in nursing home residents showed associations between polypharmacy and excessive polypharmacy and the decline in cognitive function [83]. In Japan, a study in community-dwelling older adults also found an association between polypharmacy and cognitive impairment [81]. According to a cross-sectional study in patients with newly diagnosed Parkinson’s disease, those participants with polypharmacy had significantly lower Mini-Mental State Examination scores as compared to other patients with no polypharmacy [84]. In another study investigating the associations between polypharmacy and cognitive and physical capability, polypharmacy and longstanding polypharmacy were associated with poorer cognitive capability; an even stronger negative association was observed in individuals with longstanding polypharmacy [85].

#### Polypharmacy and physical function

Polypharmacy has been shown to be associated with a physical impairment in older adults [36, 85, 86]. A systematic review on the association between polypharmacy and physical function in older adults concluded that there is a strong bidirectional relationship between polypharmacy and physical function [87]. However, a causal relationship based on the 18 observational studies included in that review could not be proven [87]. The authors concluded that objective measures of physical function and polypharmacy are necessary to prove this causal relationship in future studies [87]. Interestingly, in a multicenter study of European nursing homes polypharmacy was not associated with a faster decline in functional status [83]. Besides, disability has also been associated with polypharmacy [67, 88].

## Conclusion

The term polypharmacy is imprecise and its definition is yet subject to an ongoing debate [89]. Our findings regarding the definition of polypharmacy are in line with several previous studies [7, 11, 14, 90]. The most common definition still is the concomitant use of five or more medications.

While there was a wide range for the prevalence of polypharmacy (4–96.5%), all studies showed an increase in the prevalence of polypharmacy in older adults over time and most of them included community-dwelling older people. In addition, in almost all studies the prevalence of polypharmacy directly correlated with age. Besides, the degree of deprivation seems also to affect the prevalence of polypharmacy in older adults [44, 45]. The highest prevalence of polypharmacy (over 96.5%) was observed in older hospitalized patients in China [59]. Interestingly, the prevalence of polypharmacy among community-dwelling older adults from Korea (86.4%) appeared to be higher than in any other region of the world [24].

The imprecise and heterogenous definition of polypharmacy complicates the analysis of its prevalence and impact on relevant health outcomes. Generally, polypharmacy can only serve as an indicator for adverse clinical outcomes, a causal relation with clinical outcomes has not been unequivocally proven as prospective interventional trials on its clinical impact are largely missing. Association rather than causation has been the most prevalent outcome from studies correlating clinical outcomes and polypharmacy. The descriptive definitions of polypharmacy found in this review such as appropriate/necessary polypharmacy are potentially more useful for clinical projections. The rational use of even many drugs can be beneficial and sometimes would simply reflect the results from large randomized controlled trials, thereby limiting the role of the mere number of drugs for clinical predictions. In other words, the quality of drug treatment is crucial for a successful or beneficial therapy rather than the number of drugs used in the same patient.

Best approaches towards appropriate polypharmacy should address both over- as well as under-treatment in older patients [4, 6]. To increase the appropriateness of drug therapy at least 73 listing tools/approaches have been developed. Most of them are designed to tackle the problem of over-treatment and polypharmacy and do not address the problem of undertreatment [4]. Hence, individualized and appropriate polypharmacy is not sufficiently addressed by most of these tools. In addition, only a minority of listing tools/approaches have been validated in randomized controlled trials with relevant clinical outcomes, e.g. on physical function, hospitalization or death [4]. Of those, only the FORTA (Fit fOR The Aged) list [91] and the screening tool of older people’s prescriptions (STOPP) and screening tool to alert to right treatment (START) criteria [92] have shown a positive impact on relevant clinical endpoints in randomized controlled trials [4].

The further improvement of drug treatment in older people by interventional trials on such instruments is strongly recommended; it should lead to a better understanding of polypharmacy and of the true clinical effects of medication optimization that are not necessarily linked to the mere number of drugs.
